# DOuble SEquential External Defibrillation for Refractory Ventricular Fibrillation (DOSE VF): study protocol for a randomized controlled trial

**DOI:** 10.1186/s13063-020-04904-z

**Published:** 2020-11-26

**Authors:** Ian R. Drennan, Paul Dorian, Shelley McLeod, Ruxandra Pinto, Damon C. Scales, Linda Turner, Michael Feldman, P. Richard Verbeek, Laurie J. Morrison, Sheldon Cheskes

**Affiliations:** 1 Sunnybrook Centre for Prehospital Medicine, Sunnybrook Health Sciences Centre, 77 Brown’s Line, Suite 100, Toronto, Ontario M8W 3S2 Canada; 2 Sunnybrook Research Institute, Sunnybrook Health Sciences Centre, Toronto, Ontario Canada; 3 Li Ka Shing Knowledge Institute, St. Michael’s Hospital, Toronto, Ontario Canada; 4 St. Michael’s Hospital, Toronto, Ontario Canada; 5 Department of Family and Community Medicine, Division of Emergency Medicine, University of Toronto, Toronto, Ontario Canada; 6 Schwartz/Reisman Emergency Medicine Institute, Sinai Health System, Toronto, Ontario Canada; 7 Department of Critical Care Medicine, Sunnybrook Health Science Centre, Toronto, Ontario Canada; 8 Department of Medicine, Division of Emergency Medicine, University of Toronto, Toronto, Ontario Canada

**Keywords:** Cluster randomized controlled trial, Out-of-hospital cardiac arrest, Cardiopulmonary resuscitation, Emergency medical services, Double sequential defibrillation, Ventricular fibrillation

## Abstract

**Background:**

Despite high-quality cardiopulmonary resuscitation (CPR), early defibrillation, and antiarrhythmic medications, some patients remain in refractory ventricular fibrillation (VF) during out-of-hospital cardiac arrest. These patients have worse outcomes compared to patients who respond to initial treatment. Double sequential external defibrillation (DSED) and vector change (VC) defibrillation have been proposed as viable options for patients in refractory VF. However, the evidence supporting the use of novel defibrillation strategies is inconclusive. The objective of this study is to compare two novel therapeutic defibrillation strategies (DSED and VC) against standard defibrillation for patients with treatment refractory VF or pulseless ventricular tachycardia (pVT) during out-of-hospital cardiac arrest.

**Research question:**

Among adult (≥ 18 years) patients presenting in refractory VF or pulseless ventricular tachycardia (pVT) during out-of-hospital cardiac arrest, does DSED or VC defibrillation result in greater rates of survival to hospital discharge compared to standard defibrillation?

**Methods:**

This will be a three-arm, cluster randomized trial with repeated crossover conducted in six regions of Ontario, Canada (Peel, Halton, Toronto, Simcoe, London, and Ottawa), over 3 years. All adult (≥ 18 years) patients presenting in refractory VF (defined as patients presenting in VF/pVT and remaining in VF/pVT after three consecutive standard defibrillation attempts during out-of-hospital cardiac arrest of presumed cardiac etiology will be treated by one of three strategies: (1) continued resuscitation using standard defibrillation, (2) resuscitation involving DSED, or (3) resuscitation involving VC (change of defibrillation pads from anterior-lateral to anterior-posterior pad position) defibrillation. The primary outcome will be survival to hospital discharge. Secondary outcomes will include return of spontaneous circulation (ROSC), VF termination after the first interventional shock, VF termination inclusive of all interventional shocks, and number of defibrillation attempts to obtain ROSC. We will also perform an a priori subgroup analysis comparing rates of survival for those who receive “early DSED,” or first DSED shock is shock 4–6, to those who receive “late DSED,” or first DSED shock is shock 7 or later.

**Discussion:**

A well-designed randomized controlled trial employing a standardized approach to alternative defibrillation strategies early in the treatment of refractory VF is urgently required to determine if the treatments of DSED or VC defibrillation impact clinical outcomes.

**Trial registration:**

ClinicalTrials.gov NCT04080986. Registered on 6 September 2019.

**Supplementary information:**

The online version contains supplementary material available at 10.1186/s13063-020-04904-z.

## Background

Out-of-hospital cardiac arrest accounts for over 350,000 unexpected deaths each year in North America, nearly 100,000 of which are specifically attributable to ventricular fibrillation or pulseless ventricular tachycardia (VF/pVT) [1]. Ventricular fibrillation and pVT are considered the most treatment-responsive presentations of cardiac arrest and boast the highest rate of survival. However, despite significant advances in resuscitation treatment such as cardiopulmonary resuscitation (CPR) quality, defibrillation, and antiarrhythmic medications, some patients remain in “refractory” VF after initial therapy, usually arbitrarily defined as still in VF after three shocks. Refractory VF is associated with high mortality compared to patients with VF who respond to initial defibrillation, with survival decreasing with the number of defibrillations administered [2]. Currently, there is no additional treatment beyond continuing standard advanced cardiac life support (ACLS) that has been shown to improve survival in these patients.

Double sequential external defibrillation (DSED) has been suggested as a promising alternative to standard defibrillation for patients in refractory VF. Double sequential external defibrillation involves the use of two defibrillators, most often placed with one set of defibrillator pads in the standard anterior-lateral position and a second set in the anterior-posterior position, to deliver two shocks in rapid succession. The rationale for the use of DSED in refractory VF OHCA came from studies examining its use in atrial fibrillation [3, 4] and refractory ventricular fibrillation [5–7]. There are a number of possible mechanisms proposed for the effectiveness of DSED. These include increased energy delivery with summated shocks, the addition of an alternative vector of energy delivery (leading to improved current distribution), and decreased shock impedance leading to higher current delivery, thus increasing shock success [8].

Vector change (VC) defibrillation involving an anterior and posterior pad placement may create a higher voltage gradient in the posterior part of the ventricle, where fibrillation is most likely to restart or fail to terminate after standard pad position. The vector or pathway of flow of defibrillatory energy may also be a factor in vector change success, as shocks incorporating a pathway that includes the interventricular septum may require lower energy levels to defibrillate, and different pathways can result in increased current density (voltage gradients) in the lowest voltage areas after standard shocks [3].

Previous research examining the use of DSED for OHCA is limited to case reports and case series as well as a handful of observational studies. Cohort and case-control studies have found no difference in survival or neurological outcome when comparing the use of DSED to standard defibrillation for OHCA [9]. These studies, however, are subject to significant biases in the inclusion of patients and application of DSED that make it impossible to draw conclusions regarding the effectiveness of this strategy. Further, there is considerable heterogeneity among these studies as there is a lack of defined protocol for the use of DSED, which results in variability in DSED application and timing of first DSED shock.

It is currently not known whether DSED is superior to standard defibrillation for the treatment of refractory VF. Further, it is not known if the use of two defibrillators is superior to simply changing the defibrillation pad position (vector change or VC defibrillation) [10]. Double sequential defibrillation requires additional resources to operationalize, so it is important to determine the survival benefit of both DSED and VC compared to standard defibrillation. Therefore, the objective of this study is to compare two novel therapeutic defibrillation strategies (DSED and VC) against standard practice for patients in refractory VF during out-of-hospital cardiac arrest.

Our specific research question is, *among adult (≥ 18 years) patients with refractory VF or pulseless ventricular tachycardia (pVT) during out-of-hospital cardiac arrest, does DSED or VC defibrillation result in greater rates of survival to hospital discharge compared to standard defibrillation?* This study is reported according to SPIRIT guidance for reporting of protocols of clinical trials (Additional file 2) [11].

## Methods

### Study design and setting

This is a three-arm, crossover, cluster randomized controlled trial (RCT) comparing the effectiveness of DSED or VC defibrillation to standard defibrillation for refractory VF. Patients will be enrolled into the trial from six different regions in southern Ontario, Canada (Peel, Halton, Toronto, Simcoe, London, and Ottawa), with a mix of urban and rural centers and a combined population of 6.6 million people. Each region is served by a single municipal- or city-operated paramedic service responsible for the delivery of emergency medical care and transport. Paramedics in Ontario are certified through the Ministry of Health and Long-Term Care (MOHLTC) and operate under the medical oversight of physicians from local base hospitals. There are three base hospitals providing medical oversight to paramedics for this study: Regional Paramedic Program of Eastern Ontario (Ottawa), Sunnybrook Centre for Prehospital Medicine (Peel, Halton, Toronto, Simcoe), and Southwestern Ontario Regional Base Hospital Program (London). There are two levels of paramedics within the participating regions: primary care paramedics and advanced care paramedics. Primary care paramedics are able to provide basic life support interventions such as CPR, defibrillation, bag-valve-mask ventilation, and insertion of supraglottic airway devices. In addition to the above, advanced care paramedics are able to perform intravenous/intraosseous insertion, advanced cardiac life support medication administration, and endotracheal intubation. Cardiac arrest care is governed by provincial medical directives in accordance with the Heart and Stroke Foundation of Canada and the American Heart Association guidelines [12, 13].

### Recruitment and randomization

All adult (> 18 years) patients with refractory VF or pVT during out-of-hospital cardiac arrest of presumed cardiac etiology are eligible for inclusion in the study. Refractory ventricular fibrillation is defined as an initial presenting rhythm of ventricular fibrillation that is present on three consecutive analyses separated by 2-min intervals of CPR. While it is possible that ventricular fibrillation could terminate and re-occur during the 2 min of CPR, we feel that this definition is appropriate as pragmatically we are more interested in the rhythm status at the 2-min mark when paramedics examine the cardiac rhythm than the strict definition of “refractory” as VF never terminated, as contrasted with a transient termination of ventricular fibrillation that re-occurs prior to the next analysis. Patients suffering a traumatic cardiac arrest, patients with pre-existing do not resuscitate orders and patients in recurrent ventricular fibrillation (defined as secondary presentation of VF or those presenting in VF but did not receive three consecutive defibrillations) will be excluded. In addition, patients with initial care provided by non-participating paramedic agencies or fire services will be excluded (Table 1).

**Table 1:** Patient eligibility criteria

Inclusion criteria	Exclusion criteria
**Patients are eligible for study inclusion if,** • > 18 years of age • Presumed cardiac etiology • Presenting rhythm of VF/pVT • No ROSC or non-VF/pVT rhythm during first three analyses	**Patients are** ***not*** **eligible for inclusion if,** • Traumatic cardiac arrest • Pre-existing do not resuscitate orders • Non-VF/pVT presenting rhythm • Did not receive 3 consecutive defibrillation attempts • Care initiated by non-participating paramedic agency or fire service

Cluster randomization will occur at the level of the paramedic service in each of the six regions involved in the study. Treatment sequence generation will be performed by random computer generation by the coordinating center prior to the start of the study. Each cluster (paramedic service) will cross over every 6 months to one of the three treatment arms (standard care, DSED, VC). Over the 3 years of the trial, this means that each service will crossover a total of five times (6, 12, 18, 24, and 30 months). The randomization was performed within two blocks each containing all of the three treatment assignments and constrained that the first treatment in the second block will be different than the last treatment in the first block. Clusters will not be informed of their group assignment until necessary to make preparations to start the trial or crossover to another defibrillation strategy.

### Intervention

**Fig. 1:**
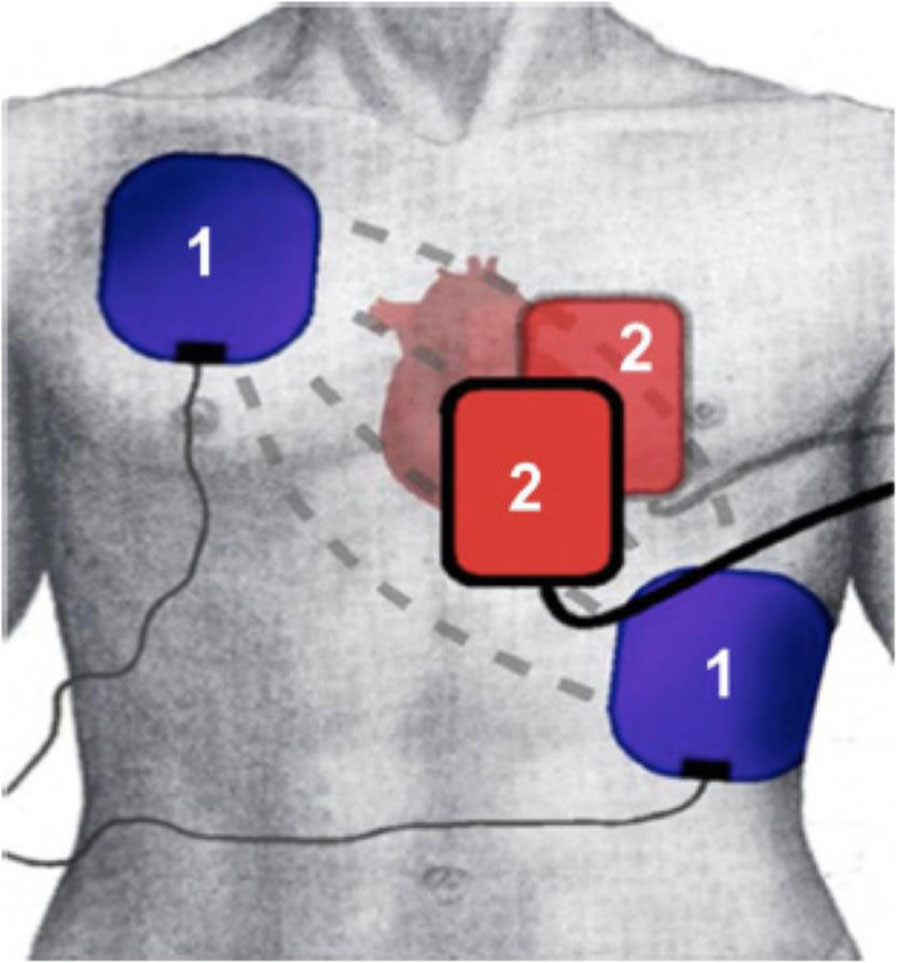
Pad placement for DSED

All included patients will have initial resuscitation performed in accordance with provincial medical directives. This includes initial CPR and defibrillation, as well as the administration of epinephrine and antiarrhythmic medication (amiodarone or lidocaine) and insertion of an advanced airway, although these interventions are not mandatory for enrollment in the study. Defibrillator pad placement will initially be in the standard anterior-lateral position as per current practice for all cardiac arrests (Fig. 1). Each rhythm analysis will occur at standard 2-min intervals.

Patients meeting the above inclusion criteria will be enrolled into the study by paramedics following the third consecutive unsuccessful defibrillation (all in standard defibrillator pad position). Following enrollment, all subsequent defibrillation attempts will be performed according to one of the three randomized intervention strategies:

1. *Standard defibrillation*: For paramedic services randomized to standard defibrillation, all subsequent defibrillation attempts will occur through pads placed in the anterior-later configuration.
2. V*ector change defibrillation*: For paramedic services randomized to vector change defibrillation, all subsequent defibrillation attempts will be delivered using anterior-posterior pad placement. Change in pad position from the initial anterior-lateral configuration will occur during the 2-min cycle of CPR following the third defibrillation, minimizing any interruptions in CPR. A new set of defibrillation pads will be used for all anterior-posterior pad placement to reduce the chance of poor adherence by re-applying the same set of pads.
3. *DSED*: For paramedic services randomized to DSED, paramedics will apply a second set of defibrillation pads in the anterior-posterior configuration (Fig. 1) Application of the second set of defibrillation pads for the second defibrillator will occur during the 2-min cycle of CPR following the third defibrillation attempt, minimizing any interruptions in CPR. All subsequent defibrillation attempts will be carried out by sequential defibrillation shocks provided by two defibrillators. To ensure that shocks are not administered at the exact same moment, we will employ a short (less than 1 s) delay to provision of the second defibrillator shock. This will be accomplished by having a single paramedic pressing the “shock” button on each defibrillator in rapid succession as opposed to simultaneously. This technique will be performed across all sites when randomized to the DSED arm to maintain consistency in application within the trial.

**Fig. 2:**
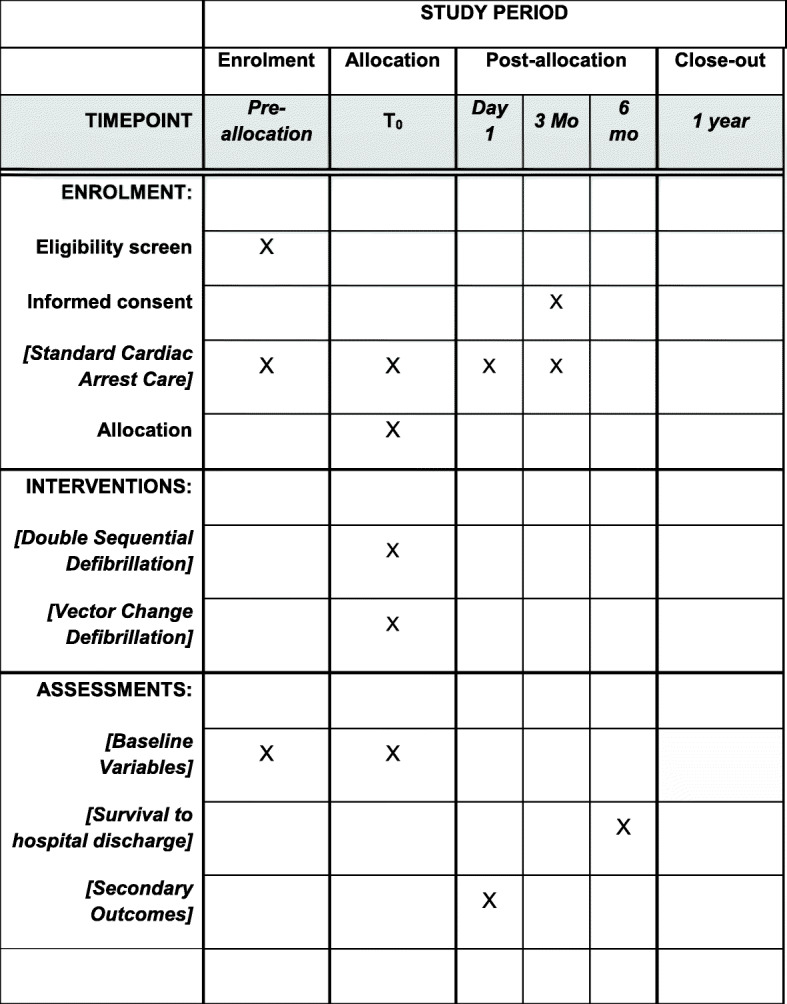
SPIRIT figure

All other prehospital treatment will follow standard treatment protocols. In-hospital resuscitation and post-cardiac arrest care will be at the discretion of the receiving hospitals. See SPIRIT figure for timeline of study milestones (Fig. 2). Study intervention adherence is monitored by study investigators on a case by case basis. Any potential issues with protocol adherence are brought to the attention of paramedics with individual feedback letters from the PI and further discussion as necessary.

### Study definitions

Ventricular fibrillation or pVT is determined by paramedic manual defibrillator analysis or semi-automatic defibrillator analysis by participating fire services, after which a shock is provided.

Termination of ventricular fibrillation is defined as the absence of fibrillation on subsequent rhythm analysis following a defibrillation and 2-min interval of CPR.

Return of spontaneous circulation is defined as any change in rhythm to an organized rhythm with a corresponding palpable pulse or blood pressure identified by paramedics on the next rhythm analysis after 2 min of CPR.

### Outcome measures

The primary outcome is survival to hospital discharge. The secondary outcomes include ROSC, termination of VF after the first interventional shock, termination of VF inclusive of all interventional shocks, and number of defibrillation attempts to obtain ROSC.

### Internal pilot

The initial phase of this study was conducted as an internal pilot aimed at examining the feasibility of performing this study in the prehospital setting prior to conducting the full RCT [8]. The pilot study was conducted with four of the six paramedic services (Peel, Halton, Simcoe, and Toronto). Feasibility outcomes were defined a priori as greater than 80% of patients receiving the correct intervention, and greater than 80% of patients receiving the intervention shock before shock six. This was predefined based on the results of our cohort study which identified improved survival with DSED administered prior to shock six [14]. The sample size for our internal pilot was based on each of the included services spending 6 months in one arm and then crossing over and enrolling patients in the second arm. This ensured that services would enroll patients in at least one intervention arm during the pilot study.

The aim of internal pilot studies is to include patients from the pilot study in the full RCT. By including these patients, internal pilots are effective strategies to enhance the efficiency of RCTs, prevent waste of valuable resources, and avoid recruitment of additional participants into a trial [15]. Conducting a pilot also allows for minor changes to be made to the trial protocol prior to continuing recruitment for the full RCT without impacting the validity of the trial as long as the changes are not substantial. The decision to move forward from the pilot study to the full RCT and to include the participants from the pilot study was made based on the identified monthly recruitment rate from each site, the feasibility of paramedics to adhere to the study protocol, and whether or not any changes to the protocol were required based on the initial recruitment. The pilot study also allowed us to re-evaluate our sample size estimation using baseline survival from our control group, and to evaluate the data linkage strategy to obtain patient vital status at hospital discharge.

The pilot study enrolled 152 patients between March 8, 2018, and September 9, 2019 [8]. Based on the results, it was felt that the pilot study met the criteria to be considered for an internal pilot (high compliance and minor adjustments to the study protocol). As such, the recruited patients are included as part of the full RCT.

We had an average of 12 patients enrolled per month among all of our sites (ranged from 1.4 to 6.1 per site). With a sixth site expected to start enrollment once the full RCT is under way, the rate of enrollment met expectations for our sample size estimations. It was further identified that 89.5% of patients were randomized to the appropriate arm, and 93.4% of patients received the proper intervention and at the proper time (prior to sixth analysis) exceeding our predefined feasibility criteria.

The following changes were made based on a thorough evaluation of the pilot study and consultation with paramedic services involved in the study:

1. Patients presenting or being in pVT during the first three rhythm analyses were included in addition to ventricular fibrillation;
2. Fire agency shocks were included in the number of defibrillation attempts a patient received prior to enrollment in the trial;
3. Any patient whose resuscitation care was started by a non-participating agency and who received one or more analyses prior to a participating agency arrival was excluded; and
4. If a second defibrillator was not available for patients randomized to DSED, the care provided remained as standard care until such time as a second defibrillator arrived and DSED could be performed.

It was felt that these changes to the study protocol were minor and that this met the definition for an internal pilot.

Finally, the pilot study allowed us to perform a linkage to administrative data for our outcome of survival at hospital discharge. We were able to successfully link data for 100% of patients that were recruited for the pilot study. We used the survival rate in our control group (standard defibrillation) to confirm our sample size estimations. Our original baseline survival estimate was 12%. This was consistent with our pilot study findings where the standard arm had a survival rate of 13.9% and, therefore, no modifications to our sample size were made. The survival rate of the intervention arms was not determined.

### Sample size requirements

The annual prevalence of paramedic-treated OHCA for the included regions is approximately 4000 of which 800 (20%) patients present in VF. Our pilot data suggest that approximately 180 patients per year meet the study criteria for refractory VF. Holmen et al. demonstrated a 30-day survival rate of 28.7% for patients receiving 1 to 3 shocks, declining to 12.4% for those receiving 4 to 10 shocks, and 4.9% for those receiving greater than 10 shocks [16]. Based on these findings, we assumed a baseline survival rate of 12% for patients who met our study criteria. This baseline survival rate was confirmed by examining the baseline survival rate of the standard defibrillation arm of the pilot RCT [8]. We hypothesize that DSED and VC defibrillation will result in higher survival compared to standard defibrillation with a minimum absolute increase of 8% in survival to hospital discharge with DSED or VC strategies compared with standard care.

Using these baseline estimates, and assuming a fixed number of EMS clusters (*n* = 6), we expect to enroll between 20 and 70 patients per cluster over 1 year. We assumed a plausible intra-cluster correlation (rho) of 0.010 and a plausible inter-period correlation (eta) of between 0.008 and 0.010 and without multiplicity correction, as has been recommended for exploratory trials involving multiple treatment arms [17, 18]. Under these conditions, the trial will have adequate power (> 80%) with an (alpha) level 0.05 to detect a minimum clinically important 9% absolute difference in survival to hospital discharge with a sample size of 310 patients per arm (total sample size of 930 patients), with approximately 150 patients from the internal pilot RCT and 780 patients from the definitive RCT.

### Data analysis

For this three-arm trial, the two treatment strategies (DSED and VC) share a common control arm (standard defibrillation). This approach allows us to maximize efficiency by comparing two new treatments to usual care in a single three-armed trial. Our primary analysis will compare DSED to standard defibrillation and VC defibrillation to standard defibrillation. Our secondary analysis will compare DSED to vector change defibrillation.

All patients will be analyzed according to randomized treatment assignment (intention-to-treat analysis). The secondary analysis will involve both a modified intention-to-treat where patients will be analyzed according to trial eligibility (patients not meeting eligibility after randomization will be excluded) and a per-protocol analysis (patients who completed the study without any major protocol violations), to account for situations where two defibrillators are not readily available in the DSED arm. We will also perform a priori subgroup analysis comparing survival for those who receive “early DSED” (DSED is applied at defibrillation attempt 4 or 6) to those who receive “late DSED” (DSED is applied at defibrillation attempt 7 or later). The primary outcome, survival to hospital discharge, will be compared across the arms of DSED and VC defibrillation as an adjusted odds ratio with 95% confidence intervals using the standard arm as a reference group. We will use a generalized linear mixed model (GLMM; logit link) with random effects for cluster-period effect and use fixed effects for cluster and for the period, to account for the effect of period on the outcome [19, 20]. The primary analysis will also adjust for the following baseline covariates known to impact outcomes after OHCA: age, sex, bystander witnessed arrest, bystander CPR provided, time to arrival of EMS, public versus private location, epinephrine, and antiarrhythmic use. For the number of defibrillation attempts to obtain ROSC, we will use a similar GLMM with log link and Poisson or negative binomial distribution depending on the distribution of the data.

### Data collection and management

#### Confidentiality and security

Data regarding patient assessments and treatment are currently collected on paramedic electronic patient care records (ePCRs). All data will be uploaded and sent to the Sunnybrook Centre for Prehospital Medicine. Data will be de-identified, and all direct identifiers (e.g., name, date of birth) corresponding to unique study ID will be stored separately and securely. All trial data will be stored in compliance with Canada’s federal privacy law, the Personal Information Protection and Electronic Documents Act 2000 [21], the Ontario privacy legislation, and PHIPA 2004, last amendment 2010 [22].

#### Data abstraction

Data sources will include paramedic ePCRs and electronic defibrillator files. Trained data abstractors will enter data from the specified files below. Data abstractors will be part of the research team and will be trained and complete all the necessary privacy and confidentiality paperwork prior to beginning data abstraction.

#### Data entry and data storage

All data collection, management, and analysis will be handled by Sunnybrook Centre for Prehospital Medicine in conjunction with Sunnybrook Health Sciences Centre. Abstracted data will be housed on a secured server at the Sunnybrook Centre for Prehospital Medicine. Only the study PI (SC), the study coordinator (ID), and statisticians (LT, RP) will have access to the final completed dataset.

#### Data linkage

The primary outcome of survival to hospital discharge will be obtained through linkage of our data with the administrative databases at Cancer Care Ontario (CCO). We expect this will occur within 6 months of the final patient enrolled. For patients who are discharged alive or transferred to a receiving hospital and subsequently discharged alive, health card numbers and other patient information as necessary will be linked at CCO with the province-wide hospital discharge abstract database (DAD) and National Ambulatory Care Reporting System (NACRS) database. Deterministic linkage will be done in cases where the health card number is available. Otherwise patient names and date of birth will be used to perform probabilistic matching.

### Data monitoring

Independent oversight of this study will be provided by a data safety monitoring board (DSMB) consisting of a biostatistician and two clinical experts unrelated to the trial. The committee will conduct a blinded interim data safety analysis to assess for lower than anticipated rates of survival between the three treatment groups. The committee will meet at 1-year intervals and provide recommendations to the steering committee. Once a total of 450 patients have been enrolled in the study, the DSMB will conduct an unblinded interim analysis to examine for sample size adjustments related to low event rates, as well as stopping the trial for harm. The decision to stop for harm will be based upon a single analysis using a Haybittle-Peto one-sided *p* value < 0.0005 as evidence for harm and criteria to stop the trial [23]. In addition, we will constantly monitor for adverse events and these will be taken into consideration during the interim analysis. The final decision to stop the trial will be made based upon the recommendations of the DSMB in discussion with the trial steering committee and the principal investigator who has the ultimate authority to stop the trial. Any modifications to the trial protocol will be communicated through writing to all relevant included parties (e.g., paramedic services, REB, and trial registries).

### Knowledge translation and dissemination of results

We have developed an integrated knowledge translation (KT) and dissemination strategy over the course of this study. During the study, we will provide regular updates to stakeholders including ongoing feedback to paramedics as well as regular presentations with updated trial status.

We will also use traditional end-of-grant KT strategies such as conference presentations, publications, and social media posts to disseminate the results of the study. Our findings will be presented at national and international conferences to a broad range of researchers, policy-makers, and end-users to ensure we target a diverse group of individuals.

## Discussion

While DSED has gained popularity among paramedic services, the evidence to date of the benefit of DSED in clinical practice is inconclusive. Previous observational studies have shown no benefit with the use of DSED for refractory ventricular fibrillation in OHCA [9]. This trial will overcome the limitations of previous observational research providing high-quality evidence for the use of DSED and VC defibrillation for OHCA.

The uncontrolled nature of prehospital medicine makes it difficult to perform rigorous, high-quality clinical trials. Our trial has a number of important design features that help to ensure its success. First, we conducted extensive pre-trial training with each paramedic involved in the study. High-quality training is essential to the running of prehospital clinical trials. Our training consisted of in-class theory and practical training as well as tailgate sessions, regular memos, posters and stickers placed throughout the service, and individual feedback to the paramedics involved in each case. Proper training, preferably by the principle investigator, can significantly increase participation by frontline paramedics leading to the overall success of the trial.

Cluster randomization at the level of the paramedic service is an important design feature for prehospital clinical research. Due to limitations in the number of personnel as well as the environment of prehospital medicine, it is often difficult for paramedics to randomize at the patient level. These challenges can reduce paramedic participation with individual patient randomization as the process becomes overly complicated to perform at the scene. Cluster randomization eliminates the need for paramedics to perform additional work to randomize patients during the call and to remember the multiple different interventions that may occur within different arms of the study. This reduces the complexity and increases paramedic satisfaction with the study. It can also reduce the time to the intervention, critical for cardiac arrest research, as paramedics know which arm of the study they are in prior to attending the call.

Use of the internal pilot during the initial phase of the study is an important design feature to maximize the efficiency of the study and to ensure patient data is not wasted. This is an important consideration for all clinical trials, especially prehospital trials, as conducting clinical research in the prehospital setting is challenging. Ensuring that efficiency is maintained in the trial by including all patients who are randomized in the final results can improve the overall success of the trial while allowing for minor modifications to be made to the trial design. One modification that was made based on the results of our pilot study that we felt was extremely important was the inclusion of shocks delivered by the fire department in the overall number of shocks provided. Previously, it has been shown that DSED is more effective if it is used earlier in a resuscitation [14]. In the pilot study, the fire department delivered a shock prior to paramedics arriving at the scene in 35% of all included cases. Exclusion of these shocks meant that the intervention shock was being unnecessarily delayed potentially biasing the study results against the intervention (towards the null). Inclusion of fire is not only more in line with the actual research question, but also provides a more accurate comparison of our intervention arms to standard defibrillation.

## Trial status

To date, more than 2500 paramedics from five of the participating paramedic services have been trained in the study protocol (Version v6; January 11, 2020). The internal pilot has been completed, and patients are currently being enrolled in the main RCT [8]. Five of the six paramedic services are currently enrolling patients (start date September 10, 2019), with the sixth service planned to start enrollment in November 2020. The pilot study enrolled 152 patients and another 95 patients have been enrolled in the main RCT. We estimate that enrollment will be completed by September 2022.

### Impact of COVID-19

As with most clinical research, enrollment in the trial was suspended from April 2020 until September 2020 due to the COVID-19 pandemic. During the initial phase of the COVID-19 pandemic, there was a drastic change in paramedic practice, including the care of cardiac arrest patients. There was an increased focus on proper personal protective equipment and reducing exposure by eliminating unnecessary personnel on scene. Due to the increased risk to paramedics and the added complexity and stress of treating cardiac arrests early in the pandemic, in consultation with our paramedic services, we decided that a temporary stop to enrollment was the most appropriate action. During the COVID-19 pandemic, survival from cardiac arrest has decreased [24, 25]. There were also concerns that the time to first shock may have increased due to the application of personal protective equipment, also potentially decreasing the frequency of VF as an initial rhythm. The COVID-19 pandemic may have also impacted the ability of paramedics to apply a second defibrillator in the DSED arm of the study.

The initial wave of COVID-19 caused considerable uncertainty regarding the treatment of OHCA patients. This uncertainty, however, decreased over time as paramedics became comfortable with donning additional personal protective equipment, and they were provided with better direction on the care of OHCA during this time. By September 2020, in consultation with paramedic services, we were able to start re-enrolling patients in the trial. At that time, Ontario was in phase 3 of re-opening with minimal (~ 100) new cases each day. Few cardiac arrests resulting from COVID-19 present in a shockable rhythm, and even fewer in refractory VF, and so these cases will not meet eligibility criteria for enrollment in the DOSE VF RCT [24–26]. Further, the management of refractory VF cardiac arrest, including transportation to hospital, has not changed for paramedics in our study regions as a result of COVID-19. Therefore, we do not anticipate that the pandemic will have a major impact on the study findings, and we are still on target to meet our enrollment numbers. We will, however, perform a sensitivity analysis to examine the results of the trial pre- and post-COVID-19.

## Supplementary Information

**Additional file 1.** Patient Consent Letter.

**Additional file 2.** Completed SPIRIT Checklist.

## Data Availability

The datasets generated and analyzed during the current study, trial protocol, and statistical code are not publicly available due to ongoing recruitment but may be available from the corresponding author on reasonable request.
