# Supplementary material for: DOuble SEquential External Defibrillation for Refractory Ventricular Fibrillation (DOSE VF): study protocol for a randomized controlled trial
Source: Trials. 2020 Nov 26;21:977. doi: 10.1186/s13063-020-04904-z (PMC7689391; doi:10.1186/s13063-020-04904-z)
Supplement: Supplementary file 1 — Additional file 1. Patient Consent Letter. [file 13063_2020_4904_MOESM1_ESM.doc]

August 29, 2026

To «patient_given_name» «patient_surname»

«street_name»

«city», «province»

«postal_code»

Dear «patient_given_name» «patient_surname» or next of kin,

**DOuble SEquential External Defibrillation for Refractory Ventricular Fibrillation- (DOSE-VF Randomized Controlled Trial)**

This letter is being sent to you from Sunnybrook Centre for Prehospital Medicine, the Base hospital affiliated with Sunnybrook Health Sciences Centre. We understand that this may be coming at a very difficult time. Please accept our apologies for this intrusion.

According to our records, **«patient_given_name» «patient_surname»** was treated by Regional Emergency Medical Services for an out-of-hospital cardiac arrest using standard procedures called Basic or Advanced Cardiac Life Support. **«patient_given_name» «patient_surname»** was included in a research study at that time, and we want you to have some information about this study, as well as contact information should you have any questions.

During a cardiac arrest, the heart does not pump blood into the brain and other important organs. As a result, survival after cardiac arrest is poor. A majority of these deaths are due to ventricular fibrillation (VF), or ‘quivering’ of the heart. One of the methods of resuscitation includes defibrillation, or administration of an electric shock, to return the heart to its normal rhythm. However, some VF patients remain in persistent or refractory VF despite defibrillation efforts. Refractory VF is defined here as patients remaining in VF after three standard defibrillation attempts.

Some studies have shown benefits to alternate strategies of defibrillation called Vector Change or Double Sequential External Defibrillation (DSED). In vector change defibrillation the pads are changed from their original position on the chest (two sticky pads on the front of the chest which are changed to one sticky pad on the front of the chest and one on the back of the chest) while DSED involves the application of two sets of defibrillator pads to the patient to provide two rapid sequential shocks, instead of the standard protocol of using one set of pads to provide a single shock. However, there is little evidence to support widespread implementation of either therapy when compared to standard therapy. It is important to note that both research strategies will be applied only after standard care has failed to return the heart to its normal rhythm.

Under usual circumstances, before enrolling a patient into a research study, consent (permission) would be obtained from the patient or their substitute decision-maker. However, in a cardiac arrest situation, since the treatment must begin immediately and the patient is not responsive, it is not possible to obtain this permission.

Before these research studies begin, they are reviewed and approved by the Research Ethics Board (REB) at Sunnybrook Health Sciences Centre. The REB’s role is to ensure that the research is scientifically sound and carried out in an ethical manner.

Mr/Mrs. «patient_surname» was enrolled into this study without consent. It was not possible to obtain consent due to the emergency nature of the situation.

We have enclosed some additional details about this study should you wish to review them. If you would like further information about this study, or would like to speak with a member of the research team, please do not hesitate to contact:

Dr. Sheldon Cheskes by email at [Sheldon.cheskes@sunnybrook.ca](mailto:Sheldon.cheskes@sunnybrook.ca) , or

Dr. Richard Verbeek by email at [Richard.verbeek@sunnybrook.ca](mailto:Richard.verbeek@sunnybrook.ca) , or

Dr. Michael Feldman by email at [Michael.Feldman@sunnybrook.ca](mailto:Michael.Feldman@sunnybrook.ca)

If you have any questions regarding the rights of research participants, you may contact the Research Ethics Board of Sunnybrook Health Sciences Centre at (416)-480-6100 ext 88144 during business hours.

Again, we apologize for this intrusion. We appreciate how very difficult this time may be for you and your family.

Sincerely,

|  |  |  |
| --- | --- | --- |
| Sheldon Cheskes, MD, CCFP (EM), FCFP  Medical Director, Peel Regional Paramedic Services and  Regional Municipality of Halton (Emergency Medical Services)  Sunnybrook Centre for Prehospital Medicine  Sunnybrook Health Sciences Centre  P. Richard Verbeek, MD, FRCPC  Medical Director, Toronto Emergency Medical Services  Sunnybrook Centre for Prehospital Medicine  Sunnybrook Health Sciences Centre  Michael Feldman, MD, FRCPC, PhD  Medical Director, Region of Simcoe Paramedic Services  Sunnybrook Centre for Prehospital Medicine  Sunnybrook Health Sciences Centre |  |  |

**DOuble SEquential External Defibrillation for Refractory Ventricular Fibrillation- (DOSE-VF Randomized Controlled Trial)**

**STUDY INVESTIGATORS:**

**Sheldon Cheskes, MD,** Principal Investigator, Sunnybrook Centre for Prehospital Medicine, Toronto, ON

**Richard Verbeek, MD,** Co-Principal Investigator, Sunnybrook Centre for Prehospital Medicine, Toronto, ON

**Michael Feldman, MD,** Co-Investigator, Sunnybrook Centre for Prehospital Medicine, Toronto, ON

**Funding:** The research program at Sunnybrook Health Sciences Centre has received funding to conduct these studies from the Heart and Stroke Foundation of Canada. The physicians involved, however, do not receive any payment for patients enrolled into the trial. The grants pay for the study-related employee salaries, contract wages and equipment costs to carry out the study.

Please note that the information below relates to the above research study in which you were enrolled. The following provides detailed information relating to this study.

You were enrolled into this study without your written consent. It was not possible to obtain your consent due to the emergency nature of the situation.

1. **BACKGROUND INFORMATION**

The investigators, in association with the Regional EMS Services, are conducting a research study to see how to best treat patients in refractory ventricular fibrillation (VF).

EMS providers treat cardiac arrests by performing cardiopulmonary resuscitation (CPR), which includes performing chest compressions and helping a person breathe. VF will be determined by paramedics and treated with administration of defibrillatory shock followed by 2 minutes of cardiopulmonary resuscitation (CPR). Patients are considered in refractory VF if they do not respond to multiple standard defibrillation attempts.

1. **WHY IS THIS STUDY BEING DONE?**

Survival after cardiac arrest is poor, and a majority of those who survive are due to VF. Despite significant advances in resuscitation efforts, there are some VF patients who remain in refractory VF. Several small studies have been performed exploring new strategies to treat those patients in refractory VF. These strategies know as vector change defibrillation and double sequential external defibrillation (DSED) have shown promising results when employed in patients in refractory VF. In patients receiving vector change defibrillation the standard pads applied to patients (two pads on the front of the chest) are changed to a position that has one pad on the front and a second pad on the back of the chest. In DSED, two sets of defibrillator pads are used to provide two rapid sequential shocks, instead of the standard protocol of using one set of pads to provide a single shock. However, there is a lack of sufficient evidence to support widespread implementation of either therapy. The DOSE-VF Pilot study is being conducted to better understand the benefits of this intervention when applied to patients who have already failed standard therapy.

1. **DESCRIPTION OF THE STUDY**

When paramedics arrived at the scene, you were treated as per the provincial protocol for treatment of patients in VF. After three consecutive standard defibrillatory attempts, you continued to remain in VF, and were randomized to continue receiving care under one of the following treatment strategies:

**Strategy 1:** You continued to receive standard care (defibrillation using one set of pads in anterior-anterior position).

**Strategy 2:** You received Vector-Change Defibrillation (defibrillation using one set of pads in anterior-posterior position).

**Strategy 3:** You received DSED (defibrillation using two defibrillators, one with anterior-posterior pad placement and the second using anterior-anterior pad placement, delivering two rapid sequential shocks).

Any medications administered, such as antiarrythmic drugs and epinephrine, continued as per current provincial standard.

Demographic information (age, gender, etc.) and health information relating to your cardiac arrest was collected from your ambulance case report form (ACR) and electronic defibrillator files.

1. **HOW MANY PATIENTS WILL TAKE OR HAVE TAKEN PART IN THE STUDIES?**

These studies are being conducted in 6 major regions in the Province of Ontario, namely the regions of Peel and Halton, the City of Toronto, the County of Simcoe and the City of Ottawa and will be enrolling patients over a three-year time frame. We expect approximately 930 patients to be included in this study during this period.

1. **WHAT ARE THE RISKS OF THE STUDY?**

Patient safety is carefully monitored and recorded for any complications of study interventions. The defibrillators used in the study have been approved by Health Canada for use in patients with VF, under the guidance of a medical professional. We will closely monitor outcomes to ensure that the interventions employed work as least as well as standard therapy.

1. **WHAT ARE THE BENEFITS OF THE STUDIES?**

You may, or may not, receive any direct benefits from being in this study. However, results from this study may further medical or scientific knowledge in the area of emergency research.

1. **PRIVACY AND CONFIDENTIALITY**

The research data will be collected from the ambulance case records and the electronic defibrillator files. All persons involved in the study, including the study investigators, coordinators, paramedics and delegates (hereby referred to as “study staff”), are committed to respecting your privacy. No other persons will have access to your personal health information without your consent, unless required by law. The study staff will make every effort to keep your personal health information private and confidential in accordance with all applicable privacy legislations, including the Personal Health Information Protection Act (PHIPA) of Ontario. The study staff will access and look at your medical records and other personal health information and collect only the information they need for the study.

Any personal identifying information (such as your name) will be “de-identified” by replacing your personal identifying information with a “unique study number”. The study coordinator and investigators are in control of the study code key, which is needed to connect your personal health information to you. The link between the study number and your personal identity will be safeguarded by the investigators.

De-identified research data may also be made available to regulatory agencies of the Canadian government. Such access will be used only for the purpose of verifying the authenticity and accuracy of the information collected for the study, without violating your confidentiality to the extent permitted by applicable laws and regulations. All information collected during this study, including your personal health information, will be kept confidential and will not be shared with anyone outside the study unless required by law. You will not be named in any reports, publications, or presentations that may come from this study.

1. **WHAT ARE THE COSTS?**

Participation in this study did not and will not result in any costs to you.

1. **PARTICIPATION AND WITHDRAWAL**

You may withdraw from further participation in this study without penalty or loss of benefits at any time after receiving this notification through contacting the study investigator. Withdrawing from the study means that information about your treatment that transpired up to the date and time of your withdrawal will be collected to maintain the scientific integrity of the study, but no further information will be collected unless required for your safety.

1. **RESEARCH ETHICS BOARD CONTACT**

If you have any questions regarding your rights as a research participant, you may contact the Research Ethics Board at Sunnybrook Health Sciences Centre at (416)-480-6100 ext. 88144 during business hours. The Research Ethics Board is a group of scientists, medical staff, and individuals from other backgrounds (including law and ethics) as well as members from the community. The committee is established by the hospital to review studies for their scientific and ethical merit.

1. **WHERE CAN I GET MORE INFORMATION?**

A description of this clinical trial is available on *http://www.ClinicalTrials.gov* (ID: NCT04080986). This web site will not include information that can identify you. At most, the web site will include a summary of the results. You can search this web site at any time. Any results from this study will be summarized in the registry or published in a confidential manner. Your name or any other identifying information will not be used in any summary or publication.

1. **STUDY CONTACT**

If you have any questions about this study or would like to receive a more detailed account of the research, please do not hesitate to contact:

Dr. Sheldon Cheskes by email at [Sheldon.cheskes@sunnybrook.ca](mailto:Sheldon.cheskes@sunnybrook.ca) , or

Dr. Richard Verbeek by email at [Richard.verbeek@sunnybrook.ca](mailto:Richard.verbeek@sunnybrook.ca)., or

Dr. Michael Feldman by email at [Michael.Feldman@sunnybrook.ca](mailto:Michael.Feldman@sunnybrook.ca)

No action on your part is required unless you wish to receive additional information or to discuss the study further.
